# Educational outcomes of CDIO-guided progressive case-based learning and gamification in undergraduate critical care nursing: an exploratory two-class quasi-experimental study

**DOI:** 10.3389/fpubh.2026.1958746

**Published:** 2026-10-09

**Authors:** Xiang Ma, Fan Yang, Aifang Yu

**Affiliations:** School of Nursing, Guilin Medical University, Guilin, Guangxi, China

**Keywords:** critical care nursing, education, gamification, nursing, baccalaureate, simulation training

## Abstract

**Background:**

This study compared educational outcomes between nursing students receiving a CDIO-guided case-based and gamified teaching program and those receiving conventional teaching.

**Materials and methods:**

This two-class quasi-experimental study included 140 nursing students from two classes between March and June 2025. Classes were assigned by lottery to the intervention (*n* = 65) or control (*n* = 75) group. The intervention integrated progressive cases, microlectures, teamwork, board games, and escape-room simulation, whereas the control received traditional lectures and Small Private Online Course self-study. Self-directed learning, critical thinking, and learning engagement were assessed before and after the course. Post-course outcomes included theoretical performance, cardiopulmonary resuscitation performance, course performance, and teaching evaluation. Analysis of covariance, Welch’s t tests, and Holm correction were applied.

**Results:**

The intervention class had higher adjusted post-course scores for self-directed learning (mean difference, 7.68; 95% CI, 3.29–12.07), critical thinking (15.27; 95% CI, 11.53–19.00), and learning engagement (7.06; 95% CI, 1.82–12.30). It also achieved higher theoretical, cardiopulmonary resuscitation, course, and teaching-evaluation scores. Students generally found the activities engaging, although some reported nervousness.

**Conclusion:**

The program was associated with improved educational outcomes, but intervention and class effects could not be separated. Multicenter studies with multiple classes per arm are required.

## Introduction

Critical care nursing education must prepare students to develop critical thinking, clinical assessment, communication, self-directed learning, and practical competence. The American Association of Colleges of Nursing identified these competencies as central components of contemporary nursing education (1). Similarly, China’s National Quality Standards for Undergraduate Education in General Higher Education Institutions, issued by the Ministry of Education in 2018, emphasized student-centered education and identified autonomous learning and critical thinking as important indicators of educational quality (2). However, conventional lecture-based teaching may provide limited opportunities for active participation, interaction, and clinical decision-making. Consequently, educational approaches such as the Conceive–Design–Implement–Operate (CDIO) model (3), case-based learning (4), simulation-based teaching (5), problem-based learning (6), and team-based learning (7) have increasingly been applied to support competency development in nursing students.

The CDIO model was originally developed in engineering education to organize learning around the processes of conceiving, designing, implementing, and operating real-world systems (8, 9). Its application in nursing education requires explicit pedagogical adaptation rather than the direct transfer of engineering terminology. In the present course, Conceive involved recognizing clinical problems, assessing available information, and identifying learning needs; Design involved planning rescue priorities and assigning team roles; Implement involved performing nursing actions and responding to evolving clinical information; and Operate involved evaluating performance, reflecting on outcomes, communicating lessons learned, and considering prevention and continuity of care. This sequence reflects the iterative nature of nursing practice by linking assessment, planning, implementation, evaluation, and reflection. Previous nursing-education studies have used CDIO-based approaches to structure active learning, clinical decision-making, and skills development (3, 10, 11). CDIO was therefore used as the organizing framework for the clinical-learning cycle, while progressive cases and gamification provided the instructional activities within that framework.

Case-based learning (CBL) is an inquiry-based approach that uses clinical cases and contextual questions to connect theoretical knowledge with clinical practice (12). In nursing education, CBL encourages active exploration, discussion, and reflection and may support the development of critical thinking and problem-solving skills (13). Numerous studies have reported improvements in theoretical knowledge, practical skills, critical thinking, self-directed learning, communication, collaboration, and learning satisfaction following CBL interventions (4, 14–16). However, conventional case teaching often presents static clinical information and may not adequately represent the evolving nature of patients’ conditions (17). Progressive case-based learning (PCL) addresses this limitation by presenting case information sequentially across different disease stages. Clinical questions, knowledge requirements, and practical tasks are introduced progressively, allowing students to reassess the situation as new information becomes available (18). This approach more closely reflects clinical practice, in which nurses frequently receive patient information incrementally. PCL has been associated with improvements in clinical reasoning, problem-solving, self-directed learning, teamwork, and satisfaction (19–22). Nevertheless, evidence regarding the effects of CBL and PCL on sustained motivation and engagement remains inconsistent, indicating a need to integrate additional active-learning strategies.

Gamification incorporates game elements into educational activities to encourage participation and support learning. Studies in health-professions education have reported potential benefits for knowledge acquisition, skills development, critical thinking, problem-solving, and learning satisfaction (23, 24). Gamified approaches may include digital simulations, virtual reality, board games, and educational escape rooms (25). A systematic review found that educational escape rooms could promote participation, motivation, and learning among health-professions students (26). Smith et al. (27) used an educational escape room with first-year nursing students and reported improvements in knowledge retention and clinical thinking. Hebert et al. (28) reported that escape-room learning supported immersive education and the development of critical thinking and clinical judgment among nursing doctoral students. Rushdan et al. (29) also found that an educational escape-room intervention was associated with improved clinical reasoning and teamwork among fourth-year nursing students. These findings suggest that gamification may complement progressive case-based learning by increasing interaction and participation.

In this study, gamification refers to the incorporation of game elements, including competition, scoring, rewards, challenges, and interactive tasks, into the Critical Care Nursing learning process. Game-based learning differs from gamification in that learning occurs through participation in an actual game structure designed around educational objectives. The present intervention incorporated both approaches: gamified elements were embedded within the CDIO-guided progressive case-based learning framework, while board-game activities and escape-room simulation provided structured game-based learning experiences. The escape-room activity was designed as a scenario-based simulation in which students applied clinical knowledge, teamwork, and decision-making skills to solve sequential tasks rather than serving as a recreational activity.

Recent nursing-education literature supports the individual components of the present teaching program, but their combined application remains insufficiently examined. Elias et al. (30) identified interactive strategies as important for promoting engagement in nursing education. Vázquez-Calatayud et al. (31) reported that integrating real-world and game-based learning could support decision-making, while Mackavey et al. (32) described interactive case discussion and game-informed activities as strategies for increasing engagement and knowledge synthesis. However, limited evidence is available on the integration of progressive cases, gamified activities, and simulation within a CDIO-organized undergraduate critical care nursing course. The present study therefore evaluated the integrated teaching program as a whole rather than attempting to determine the effects of its individual components.

This study examined a redesigned critical care nursing course delivered before students entered their clinical internship. The intervention integrated progressive cases, pre-class microlearning, structured teamwork, board-game review, and escape-room simulation within an explicitly mapped CDIO cycle. The main exploratory objective was to compare baseline-adjusted post-course self-directed learning, critical thinking, and learning-engagement scores between two intact classes. Additional exploratory objectives were to compare theoretical performance, cardiopulmonary resuscitation performance, overall course performance, and teaching evaluation and to summarize students’ open-ended feedback descriptively. It was expected that the intervention class would demonstrate higher baseline-adjusted post-course self-directed learning, critical thinking, and learning-engagement scores than the control class. Given the inclusion of only one class in each study arm, the study was intended to generate preliminary evidence for future evaluations involving multiple classes per condition.

## Methods

### Study design

This prospective, exploratory, two-class quasi-experimental study included descriptive open-ended feedback. Two intact classes were assigned at the class level by lottery, with one class receiving the intervention and the other receiving the control condition. The intact class, rather than the individual student, was the unit of assignment. The study was reported in accordance with the TREND statement for nonrandomized evaluations of behavioral and educational interventions.

### Setting and participants

The study was conducted from March to June 2025 among third-year undergraduate nursing students at Guilin Medical University. The two available intact classes constituted a convenience sample. One class was assigned by class-level lottery to the intervention condition (*n* = 65), and the other was assigned to the control condition (*n* = 75). Both classes completed the semester-long Critical Care Nursing course.

The inclusion criteria were: (a) enrollment as a full-time undergraduate nursing student; (b) first-time enrollment in the Critical Care Nursing course; and (c) voluntary participation in the study. Students who took a leave of absence, withdrew from the course, or were absent from three or more classes were excluded. All 140 eligible and consenting students completed the course and follow-up assessments and were included in the analysis.

### Sample size

No *a priori* sample-size calculation was performed because the sample size was determined by the number of eligible students in the two available intact classes. Because only one class was included in each study arm, the analyses were considered exploratory.

### Ethical considerations

The study was approved by the Ethics Committee of Guilin Medical University (Approval No. GLMC20250305). All participants were informed about the study objectives and procedures, and written informed consent was obtained before participation.

### Intervention and control conditions

Both classes used the same textbook, *Critical Care Nursing*, 5th edition, and followed the same course syllabus. Course content was delivered by an experienced instructor. The control class received conventional lectures supplemented by Small Private Online Course (SPOC) self-study. Practical sessions followed a lecture–demonstration–group practice–review format.

The intervention class received a CDIO-guided teaching program integrating progressive case-based learning and gamification. The program included pre-class microlectures, progressive clinical cases, structured teamwork, board-game activities, escape-room simulation, individual skills assessment, group reflection, and instructor feedback. The intervention consisted of three integrated components: (1) progressive case-based learning as the clinical reasoning framework, (2) gamified learning elements such as scoring, competition, and rewards, and (3) escape-room simulation activities involving scenario-based clinical challenges.

Both groups completed the same Critical Care Nursing course during the same semester with equivalent scheduled instructional exposure. The intervention group received CDIO-guided progressive case-based learning integrated with gamified activities within the existing course schedule, whereas the control group received conventional teaching. Therefore, the intervention represented a difference in instructional strategy rather than additional student contact time.

The intervention class received a CDIO-guided teaching program integrating progressive case-based learning and gamification. The program included pre-class microlectures, progressive clinical cases, structured teamwork, board-game activities, escape-room simulation, individual skills assessment, group reflection, and instructor feedback (Supplementary Table S4).

### Teaching team and program development

The teaching team comprised 16 members: one department head, seven full-time faculty members, and eight clinical nurses from the Emergency Department or Intensive Care Unit. The team developed the teaching objectives, lesson plans, case materials, and gamified activities and conducted monthly collective lesson-planning sessions. A professor with a doctoral degree in education and more than 20 years of clinical and teaching experience served as a teaching consultant and provided guidance during program development and implementation.

The instructors involved in course delivery followed the same course objectives, syllabus, assessment requirements, and collectively developed teaching plans to standardize implementation across the two classes. The same teaching team was responsible for course delivery in both classes, and instructors followed common course objectives, syllabus, assessment requirements, and collectively developed teaching plans to standardize implementation.

Teaching objectives were developed with reference to the National Standards for Undergraduate Nursing Education and Bloom’s Taxonomy. The objectives covered three domains: (a) knowledge, including basic first aid knowledge and emergency procedures for common critical conditions; (b) competencies, including first aid skills, critical thinking, and self-directed learning; and (c) professional values, including learning motivation, professional responsibility, and humanistic care.

The progressive case-based and gamified teaching program was organized according to the CDIO framework. An initial teaching plan was developed through a literature review and alignment with the course objectives. Five nursing-education experts reviewed and refined the plan, which was then finalized through teaching-team discussions (Supplementary Table S1).

### Intervention implementation

The intervention followed a three-stage clinical framework comprising disease identification, disease rescue, and disease prevention. Progressive cases and gamified activities were organized across the four CDIO phases: Conceive, Design, Implement, and Operate. Detailed descriptions of the progressive case scenarios, student challenges, learning objectives, game implementation process, and instructor feedback procedures are provided in Supplementary Table S3. Acute organophosphorus pesticide poisoning is presented as an example of the teaching process. Progressive cases and gamified activities were organized across the four CDIO phases (Supplementary Table S4).

During the Conceive phase, which occurred before class, teachers prepared microlecture videos addressing relevant theoretical knowledge and practical procedures. Course materials were uploaded to the online learning platform 1 week before class for student preparation.

During the Design phase, conducted in the theoretical class, students formed first aid teams and participated in gamified activities, including board-game quizzes (Supplementary Table S2). Teachers reviewed areas of weak performance identified during the games and used progressive cases to guide students in developing rescue plans through group discussion and inquiry-based learning.

Students participated in emergency response teams consisting of five members. Before each gamified activity, instructors introduced the clinical scenario, learning objectives, activity rules, and scoring criteria. Students then collaborated to analyze clinical information, solve sequential scenario-based tasks, and complete emergency response challenges. The escape-room simulation consisted of multiple stages, with progression dependent on successful completion of predefined tasks. During the activities, instructors monitored student performance and provided guidance when necessary. After completion, students participated in group reflection and instructor feedback to consolidate clinical reasoning and knowledge application.

During the Implement phase, conducted in the laboratory class, students participated in a three-stage escape-room simulation covering poison identification, poisoning rescue, and poisoning prevention. Teams completed scenario-based tasks, performed key first aid procedures, interpreted evolving clinical information, and managed simulated emergencies with instructor guidance.

During the Operate phase, students completed individual practical-skills assessments, participated in group reflection and presentations, and received peer and instructor feedback. Certificates and small gifts were awarded to groups and individuals demonstrating high performance.

The progressive cases followed a scenario–problem–knowledge-and-skills structure and were used in both theoretical and laboratory sessions. Case information was presented sequentially to reflect changes in the patient’s condition and to require repeated assessment and decision-making. The progressive-case activity lasted approximately 40 min (Supplementary Table S3).

Following completion of the 40-min escape-room simulation, a structured instructor-led reflection session was conducted. Students discussed their clinical reasoning processes, teamwork experiences, challenges encountered during task completion, and possible improvements in emergency response strategies. The session was facilitated by members of the teaching team, who provided feedback, reinforced key knowledge points, and addressed identified learning gaps.

The implementation of key intervention components was monitored according to the predefined teaching plan, including CDIO integration, progressive case-based learning, gamified activities, escape-room simulation, instructor feedback, and reflection activities (Supplementary Table S5).

### Outcome measures

Self-directed learning, critical thinking, and learning engagement were assessed before and after the course. Theoretical examination performance, cardiopulmonary resuscitation performance, overall course performance, and teaching evaluation were assessed after completion of the course. Open-ended feedback was collected from students in the intervention class.

### Self-directed learning ability

Self-directed learning was assessed using the Nursing Students’ Autonomous Learning Ability Scale, originally developed by Cheng et al. (33) and subsequently adapted by Defang (34). The scale contains 20 items across four dimensions: learning motivation (6 items), planning and implementation (6 items), self-management (4 items), and interpersonal communication (4 items). Items are rated on a 5-point Likert scale ranging from 1 to 5. Total scores range from 20 to 100, with higher scores indicating greater self-directed learning ability. Cronbach’s alpha for the scale in the present study was 0.847.

### Critical thinking

Critical thinking was assessed using the simplified Critical Thinking Scale developed by Peng et al. (35) and subsequently revised by Yang et al. (36) The 31-item instrument includes six dimensions: truth-seeking, analytical ability, systematic ability, self-confidence, curiosity, and maturity. Items are rated on a 6-point Likert scale. Negatively worded items in the truth-seeking and maturity dimensions are reverse-scored. Total scores range from 31 to 186, with higher scores indicating stronger critical thinking. Cronbach’s alpha in the present study was 0.765.

### Learning engagement

Learning engagement was assessed using the College Students’ Learning Engagement Questionnaire developed by Youguo et al. (37) The questionnaire contains 20 items across three dimensions: behavioral engagement (6 items), cognitive engagement (7 items), and emotional engagement (7 items). Items are rated on a 5-point Likert scale ranging from 1 to 5. Total scores range from 20 to 100, with higher scores indicating greater learning engagement. Cronbach’s alpha in the present study was 0.875.

### Theoretical and cardiopulmonary resuscitation performance

After completing the course, students in both classes completed a standardized theoretical examination developed by the same teaching team and approved by the department head and the college’s vice dean for teaching. The examination had a maximum score of 100 points. Multiple-choice questions accounted for 40 points, with 1 point awarded for each correct answer, and clinical case-analysis questions accounted for 60 points, with individual questions valued at 6–10 points.

Students in both classes also completed an individual cardiopulmonary resuscitation (CPR) performance assessment during the laboratory component of the course. Students were assessed using the same scoring criteria, with a maximum score of 100 points. The intervention class completed the assessment after the team-based gamified activities, whereas the control class completed it during the final laboratory session.

The overall course score was calculated using 70% of the theoretical examination score and 30% of the CPR performance score. CPR was selected as the practical outcome because it is a fundamental, time-sensitive critical care nursing skill involving psychomotor performance and the application of clinical judgment under pressure.

### Teaching evaluation

Teaching evaluation was assessed using a self-developed 10-item questionnaire informed by relevant literature (38). The questionnaire assessed the perceived necessity and sufficiency of teaching materials and resources, clarity and comprehensibility of materials, appropriateness of content difficulty, support for knowledge application, promotion of reasoning and critical thinking, integration of theory with clinical practice, perceived improvement in clinical-practice skills, and confidence in initiating rescue actions in a similar clinical situation.

Each item was rated on a 5-point Likert scale ranging from strongly disagree (1) to strongly agree (5). Total scores ranged from 10 to 50, with higher scores indicating a more favorable teaching evaluation. Cronbach’s alpha for the questionnaire in the present study was 0.962. Comparisons of the individual questionnaire items were considered exploratory.

### Open-ended feedback

Students in the intervention class completed three self-developed open-ended questions concerning their learning experiences and emotional responses during the gamified activities, suggestions for improving the activities, and opinions regarding the use of the approach in other subjects (Supplementary File 1).

The responses were used as descriptive feedback rather than as a formal qualitative research component. The research team reviewed the responses and grouped recurring content into descriptive, non-mutually exclusive categories. Counts were calculated using the total intervention-class sample as the denominator (*n* = 65). A single response could contribute to more than one category; therefore, category counts were not expected to sum to 65. No claims of thematic saturation or formal thematic analysis were made.

### Data collection

Self-directed learning, critical thinking, and learning engagement were measured before the course and again after completion of the course. The theoretical examination, CPR performance assessment, and teaching-evaluation questionnaire were administered after the course. Open-ended feedback was collected from the intervention class following completion of the gamified teaching activities. No outcome data were missing.

### Statistical analysis

Statistical analyses were conducted using IBM SPSS Statistics, version 23.0. Continuous variables were summarized using means and standard deviations, and categorical variables were summarized using frequencies and percentages. Baseline participant characteristics were examined using Welch’s t test or the chi-square test, as appropriate.

For self-directed learning, critical thinking, and learning engagement, exploratory analysis of covariance models were used. In each model, the post-course score was entered as the dependent variable, study class was entered as the factor, and the corresponding pre-course score was entered as a covariate. Tests of the homogeneity-of-regression-slopes assumption did not identify statistically significant interactions between study class and baseline score for any of the three outcomes (all *p* > 0.10).

Post-course-only continuous outcomes were compared using Welch’s t tests. Analysis of covariance results were reported as adjusted mean differences with 95% confidence intervals and partial eta squared. Post-course-only comparisons were reported as unadjusted mean differences with 95% confidence intervals and Hedges’ *g*.

Holm’s step-down procedure was applied across the 17 inferential comparisons reported in the outcome tables. Both unadjusted and Holm-adjusted *p* values were presented. All statistical tests were two-sided, and statistical significance was set at *p* < 0.05. Because there were no missing outcome data, no imputation was performed.

Assignment occurred at the class level, and only one class was included in each study arm. Consequently, the teaching condition was completely confounded with class. Multilevel models or cluster-robust standard errors could not provide a separately identifiable intervention effect with one class per condition. The individual-level analyses were therefore considered exploratory and did not account for between-class heterogeneity.

## Results

The structure of the CDIO-guided teaching program, the gamified teaching plan, and the progressive case design are presented in Supplementary Tables S1–S3, respectively. Supplementary Table S1 summarizes the teaching objectives, content, methods, evaluation procedures, and teaching stages organized according to the CDIO framework. Supplementary Table S2 describes the gamified activities and scoring procedures, while Supplementary Table S3 presents the progressive clinical case used to illustrate the intervention.

### Participant characteristics and flow

Two intact third-year nursing classes comprising 140 students were assessed for eligibility. All eligible students consented and were enrolled. Following class-level allocation by lottery, 65 students received the intervention condition and 75 received the control condition. All participants completed their assigned course condition and follow-up assessments, with no losses to follow-up. All 140 participants were included in the analysis, as shown in Figure 1.

**Figure 1:**
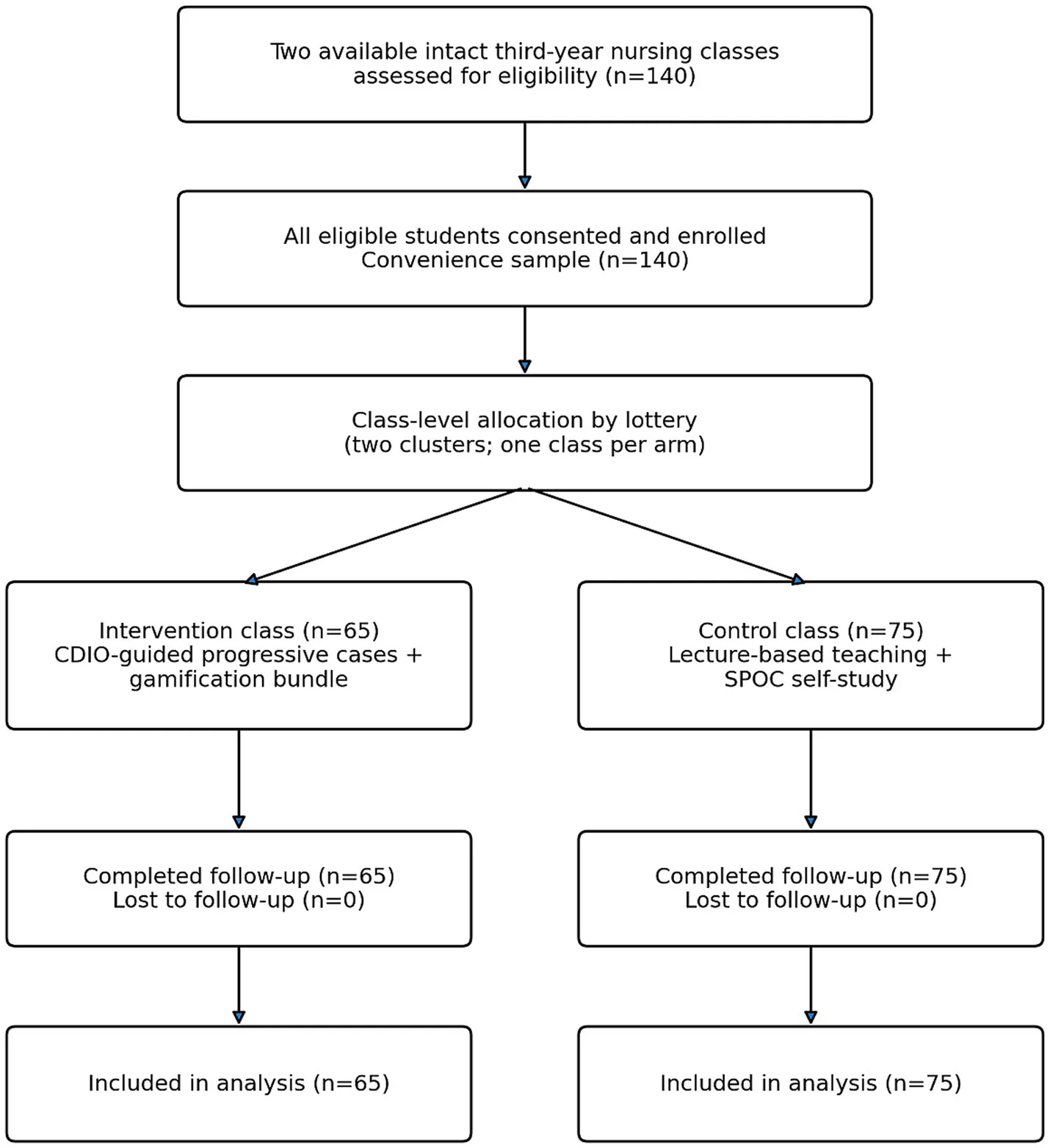
Participant flow through the two-class quasi-experimental study.

The participants included 39 male students (27.9%) and 101 female students (72.1%). Their ages ranged from 18 to 23 years, with a mean age of 20.22 ± 0.81 years. All participants had completed the first five semesters of general and nursing-related courses. No statistically significant between-class differences were observed in age, sex, previous awareness of gamification, previous experience with gamified teaching, or examination scores from the previous semester. Participant characteristics are presented in Table 1.

**Table 1:** Participant characteristics by study class.

Characteristic	Intervention class (*n* = 65)	Control class (*n* = 75)	*t* or *χ*^2^	*p*
Age	20.18 ± 0.81	20.25 ± 0.81	−0.502^a^	0.616
Sex			3.42^b^	0.064
Male	23 (35.4)	16 (21.3)		
Female	42 (64.6)	59 (78.7)		
Previous awareness of gamification, *n* (%)			0.21^b^	0.646
Yes	44 (67.7)	48 (64.0)		
No	21 (32.3)	27 (36.0)		
Previous experience with gamified teaching, *n* (%)			0.85^b^	0.356
Yes	38 (58.5)	38 (50.7)		
No	27 (41.5)	37 (49.3)		
Previous-semester examination score	70.89 ± 7.79	69.93 ± 7.98	0.72^a^	0.472

Data are presented as mean ± standard deviation or *n* (%). Percentages were calculated within each study class.

a *t* value = Welch’s *t*.

b *χ*^2^ value = Chi-square.

### Self-directed learning, critical thinking, and learning engagement

Pre- and post-course scores and exploratory baseline-adjusted comparisons are presented in Table 2. After adjustment for the corresponding baseline score, the intervention class had a higher post-course self-directed learning score than the control class (adjusted mean difference = 7.68, 95% CI = 3.29–12.07; partial η^2^ = 0.080; Holm-adjusted *p* = 0.009).

**Table 2:** Pre- and post-course outcomes and exploratory baseline-adjusted comparisons.

Outcome	Time	Intervention class (*n* = 65), mean ± SD	Control class (*n* = 75), mean ± SD	Adjusted mean difference (95% CI)	*F*(1,137)	Partial η^2^	*p*	Holm-adjusted *p*
Self-directed learning	Pre	65.89 ± 12.91	68.99 ± 13.72					
	Post	74.48 ± 14.79	66.95 ± 11.20	7.68 (3.29–12.07)	11.96	0.08	<0.001	0.009
Critical thinking	Pre	108.98 ± 16.28	107.73 ± 23.20					
	Post	128.29 ± 11.36	113.00 ± 10.87	15.27 (11.53–19.00)	65.41	0.323	<0.001	<0.001
Learning engagement	Pre	62.37 ± 17.99	59.65 ± 13.49					
	Post	69.65 ± 17.94	62.69 ± 13.09	7.06 (1.82–12.30)	7.1	0.049	0.009	0.034

ANCOVA models included the post-course score as the dependent variable, study condition as the factor, and the corresponding pre-course score as the covariate. Positive adjusted differences favor the intervention class. Holm adjustment was applied across the 17 comparisons reported in Tables 2–4. Because one intact class was included in each study condition, the individual-level analyses were exploratory and did not account for between-class heterogeneity. ANCOVA, analysis of covariance; CI, confidence interval; SD, standard deviation.

The intervention class also had a higher post-course critical-thinking score (adjusted mean difference = 15.27, 95% CI = 11.53–19.00; partial η^2^ = 0.323; Holm-adjusted *p* < 0.001) and learning-engagement score (adjusted mean difference = 7.06, 95% CI = 1.82–12.30; partial η^2^ = 0.049; Holm-adjusted *p* = 0.034).

### Post-course academic and CPR performance

Post-course academic and cardiopulmonary resuscitation performance comparisons are presented in Table 3. The intervention class had a higher CPR performance score than the control class, with a mean difference of 1.95 points (95% CI = 0.74–3.16; Hedges’ *g* = 0.54; Holm-adjusted *p* = 0.016).

**Table 3:** Post-course performance comparisons with effect estimates.

Outcome	Intervention class (*n* = 65), mean ± SD	Control class (*n* = 75), mean ± SD	Mean difference (95% CI)	Welch’s *t*	*df*	Hedges *g*	*p*	Holm-adjusted *p*
CPR performance score	85.17 ± 3.56	83.22 ± 3.65	1.95 (0.74–3.16)	3.2	136	0.54	0.002	0.016
Theoretical examination score	73.62 ± 6.91	70.09 ± 5.27	3.53 (1.45–5.61)	3.35	118.6	0.58	0.001	0.011
Overall course score	77.09 ± 5.09	74.03 ± 4.19	3.05 (1.48–4.63)	3.84	124.2	0.66	<0.001	0.003

Positive mean differences favor the intervention class. Holm adjustment was applied across the 17 comparisons reported in Tables 2–4. CI, confidence interval; CPR, cardiopulmonary resuscitation; df, degrees of freedom; SD, standard deviation.

The intervention class also had a higher theoretical examination score (mean difference = 3.53, 95% CI = 1.45–5.61; Hedges’ *g* = 0.58; Holm-adjusted *p* = 0.011) and overall course score (mean difference = 3.05, 95% CI = 1.48–4.63; Hedges’ *g* = 0.66; Holm-adjusted *p* = 0.003). Although the standardized difference in CPR performance was moderate, the absolute difference was approximately 2 points on a 100-point scale.

### Teaching evaluation

Teaching-evaluation outcomes are presented in Table 4. The intervention class had a higher total teaching-evaluation score than the control class (mean difference = 4.51, 95% CI = 1.89–7.12; Hedges’ *g* = 0.58; Holm-adjusted *p* = 0.011).

**Table 4:** Post-course teaching-evaluation comparisons with effect estimates.

Item	Intervention class (*n* = 65), mean ± SD	Control class (*n* = 75), mean ± SD	Mean difference (95% CI)	Hedges *g*	*p*	Holm-adjusted *p*
1. Teacher-provided information was necessary	3.88 ± 1.07	3.49 ± 0.92	0.38 (0.05–0.72)	0.38	0.026	0.06
2. Exercises improved reasoning and critical thinking	3.71 ± 0.96	3.35 ± 0.83	0.36 (0.06–0.66)	0.4	0.02	0.06
3. Confidence in taking rescue action in a similar clinical situation	3.74 ± 0.92	3.23 ± 0.86	0.51 (0.21–0.81)	0.57	0.001	0.011*
4. Teaching activities linked theory with clinical practice	3.80 ± 0.94	3.35 ± 0.83	0.45 (0.15–0.75)	0.51	0.003	0.02*
5. Activities improved application of clinical-practice skills	3.82 ± 0.95	3.35 ± 0.86	0.47 (0.17–0.77)	0.52	0.003	0.02*
6. Content difficulty was acceptable	3.75 ± 0.97	3.24 ± 0.69	0.51 (0.23–0.80)	0.61	<0.001	0.008*
7. Teacher provided sufficient resources	3.75 ± 0.90	3.29 ± 0.88	0.46 (0.16–0.76)	0.51	0.003	0.02*
8. Materials were easy to understand	3.78 ± 0.96	3.27 ± 0.76	0.52 (0.22–0.81)	0.6	<0.001	0.009*
9. Teacher explanations supported application of knowledge	3.77 ± 0.98	3.44 ± 0.78	0.33 (0.03–0.63)	0.37	0.031	0.06
10. Teacher-provided materials were clear and accurate	3.80 ± 0.99	3.29 ± 0.90	0.51 (0.19–0.83)	0.54	0.002	0.016*
Total teaching-evaluation score	37.80 ± 8.58	33.29 ± 6.78	4.51 (1.89–7.12)	0.58	0.001	0.011*

Items were rated from 1 (strongly disagree) to 5 (strongly agree); total scores ranged from 10 to 50, with higher scores indicating a more favorable teaching evaluation. Positive mean differences favor the intervention class. Holm adjustment was applied across the 17 comparisons reported in Tables 2–4. Items 1, 2, and 9 were not statistically significant after Holm adjustment. CI, confidence interval; SD, standard deviation. *Significant values *p* < 0.05.

After Holm adjustment across the comparisons reported in Tables 2–4, seven of the ten individual teaching-evaluation items remained statistically significant at *p* < 0.05. Items 1, 2, and 9 were not statistically significant after adjustment.

### Descriptive feedback from the intervention class

Open-ended feedback from the 65 students in the intervention class was summarized using non-mutually exclusive descriptive categories. Forty-one students (63.1%) described the activities as engaging, 27 (41.5%) reported nervousness during gameplay, 12 (18.5%) described the activities as thrilling, and eight (12.3%) stated that the games helped them maintain focus. Two students (3.1%) mentioned tiredness or boredom and indicated that they did not want the approach extended to other subjects.

Suggested improvements included greater variety in game formats (*n* = 10, 15.4%), longer activity duration (*n* = 8, 12.3%), and clearer or simpler rules (*n* = 8, 12.3%). Because one response could contribute to multiple categories, the counts and percentages overlap and should not be summed. The open-ended questionnaire is provided in Supplementary File 1.

## Discussion

### Self-directed learning, critical thinking, and learning engagement

Self-directed learning is important for lifelong professional development (39), while critical thinking is a central competency in nursing education (1). Both abilities are also recognized as indicators of educational quality in undergraduate nursing programs (2). In this study, the intervention class had higher baseline-adjusted post-course self-directed learning and critical-thinking scores than the control class. These findings are consistent with previous studies examining CDIO-based education and progressive case-based learning (3, 19). However, because only one intact class was included in each condition, the observed differences should be interpreted as exploratory between-class associations rather than definitive intervention effects.

Several features of the teaching program may help explain the findings. First, the CDIO framework organized learning around problem identification, planning, implementation, evaluation, and reflection, which may have encouraged students to connect theoretical knowledge with clinical tasks. Second, pre-class microlearning and progressive cases required students to review information, identify learning needs, and respond to new clinical information as the case developed. Third, the board-game and escape-room activities may have increased participation and provided repeated opportunities to apply knowledge. Nevertheless, the relative contribution of these components cannot be determined because they were delivered as an integrated teaching bundle.

The critical-thinking result requires particular caution. The mean score increased from 108.98 to 128.29 in the intervention class and from 107.73 to 113.00 in the control class. The post-course standardized difference was large and substantially greater than those observed for most other outcomes. A change of this magnitude over one semester may reflect genuine learning together with measurement reactivity, novelty, expectancy or demand effects, differential engagement with the self-report instrument, and unmeasured class-level influences. The absence of blinded assessment and reliance on self-reported critical thinking further limit interpretation. Future studies should use multiple classes per condition, objective or blinded assessments where feasible, and longer-term follow-up to determine whether the observed difference is reproducible and sustained.

Learning engagement contributes to academic achievement and continuing professional development (40). Participatory and interactive learning approaches have been associated with greater engagement and richer learning experiences in nursing education (41). In the present study, pre-class preparation, progressive case analysis, collaborative problem-solving, and gamified activities may have encouraged students to devote greater attention and effort to course tasks. However, because engagement was self-reported and the study included one class per condition, the result should be interpreted as an exploratory between-class association.

### Post-course academic and CPR performance

The intervention class had higher theoretical examination, CPR performance, and overall course scores than the control class. The standardized differences were moderate for all three outcomes. However, the absolute difference in CPR performance was approximately 2 points on a 100-point scale, indicating that statistical effect sizes should be considered together with the practical magnitude of the score difference.

Progressive cases may have supported students in connecting theoretical concepts with evolving clinical information, while gamified tasks may have encouraged active participation and repeated practice. These mechanisms remain possible explanations rather than confirmed causal pathways. The two-class design prevents the observed differences from being attributed specifically to the teaching condition rather than to cohort composition, class culture, instructor–class interaction, or other class-level factors.

CPR was selected as the practical outcome because it is a fundamental and time-sensitive critical care nursing skill. Nevertheless, CPR performance represents only one aspect of clinical competence and does not fully assess broader abilities such as recognizing patient deterioration, prioritizing nursing problems, integrating complex information, and managing changing clinical situations. Future evaluations should therefore include more comprehensive simulation-based assessments of clinical reasoning and decision-making.

The intervention incorporated the CDIO framework, progressive cases, microlectures, structured teamwork, board games, and escape-room simulation. Consequently, the contribution of any individual component cannot be isolated. Differences in time spent on learning activities, instructor attention, or instructor enthusiasm may also have influenced the outcomes because these factors were not formally measured.

### Teaching evaluation and student feedback

Teaching-evaluation scores were more favorable in the intervention class, with a moderate standardized difference in the total score. Progressive cases and gamified activities may have increased the perceived relevance of course content by connecting theoretical knowledge with simulated clinical tasks. However, the teaching-evaluation questionnaire was self-reported and self-developed, and responses may have been influenced by novelty, expectancy, or demand characteristics.

Open-ended feedback provided additional descriptive information about students’ experiences. Engagement was the most frequently reported positive response, although nervousness during gameplay was also common. Only two students reported tiredness or boredom and indicated that they did not want the approach applied to other subjects; this small number should not be overinterpreted. The more frequent recommendations concerned greater variety in game formats, longer activity duration, and clearer or simpler rules. These findings suggest that future implementation should balance challenge with clarity, provide adequate activity time, and vary the game format to reduce repetition.

### Implications for nursing education

Progressive cases may provide a useful structure for presenting clinical information in stages, requiring students to reassess priorities as a patient’s condition evolves. Gamified activities may complement this process by supporting participation, teamwork, and repeated practice. However, the present findings are preliminary and do not justify routine implementation without further evaluation.

Nursing programs considering a similar approach should account for the number of instructors required, preparation time, simulation equipment, classroom space, and the organization of student teams. Instructions and scoring rules should be clearly explained, and the duration and difficulty of activities should be adjusted to students’ level of preparation. Pilot implementation across several classes may help identify logistical problems and determine whether positive educational outcomes can be reproduced before the approach is adopted more broadly.

### Limitations

This study has several limitations. First, class-level allocation was used, but only one intact class was included in each study arm. The teaching condition was therefore completely confounded with class. Observed differences may reflect cohort composition, timetable effects, peer culture, instructor–class interaction, or other class-level characteristics rather than the intervention itself. With only one class per arm, valid cluster-adjusted treatment inference was not possible, and the reported confidence intervals may underestimate uncertainty arising from between-class heterogeneity.

Second, the study was conducted in a single nursing program, which limits the generalizability of the findings. The principal sampling limitation was the small number of classes rather than the number of individual students. Third, participants and instructors were not blinded, and assessor blinding was not reported. Expectancy, novelty, and demand effects may therefore have influenced performance and questionnaire responses.

Fourth, self-directed learning, critical thinking, learning engagement, and teaching evaluation were self-reported. In addition, the teaching-evaluation questionnaire was self-developed and had limited evidence of validity beyond internal consistency in the present sample. Fifth, total time on task, instructor attention, instructor enthusiasm, and possible exchanges of course materials between the two classes were not formally measured.

Sixth, outcomes were assessed immediately after the course, and no longer-term follow-up was conducted. It is therefore unclear whether the observed differences were sustained. Seventh, the multicomponent intervention prevents identification of the effects of individual elements. Finally, the timing of the CPR assessment differed between the classes, with the intervention class assessed after the team-based activities and the control class assessed during the final laboratory session. This difference may have influenced CPR performance and should be considered when interpreting the findings.

Future studies should include multiple classes in each condition, prespecify primary outcomes, use objective or blinded assessment where feasible, standardize assessment timing, measure intervention fidelity and time on task, and include longer-term follow-up. Multicenter studies would also help determine whether the findings are reproducible across different nursing-education settings.

## Conclusion

In this exploratory two-class study, the CDIO-guided progressive case-based and gamified teaching program was associated with higher self-directed learning, critical-thinking, learning-engagement, academic-performance, and teaching-evaluation scores. However, because only one intact class was included in each study arm, class effects could not be separated from the teaching condition, and the findings should be considered preliminary. The multicomponent intervention also prevents the identification of the contribution of individual teaching elements. Future multicenter studies should include multiple classes per arm, standardized or blinded assessments, and longer-term follow-up.

## Data Availability

The raw data supporting the conclusions of this article will be made available by the authors, without undue reservation.
