# Supplementary material for: Educational outcomes of CDIO-guided progressive case-based learning and gamification in undergraduate critical care nursing: an exploratory two-class quasi-experimental study
Source: Front Public Health. 2026 Oct 9;14:1958746. doi: 10.3389/fpubh.2026.1958746 (PMC13690949; doi:10.3389/fpubh.2026.1958746)
Supplement: Supplementary file 1 [file Supplementary_file_1.docx]

**Supplementary File 1**

**Open-Ended Questionnaire on Gamified Teaching in the Critical Care Nursing Course**

**Purpose**

This self-developed open-ended questionnaire was used to collect qualitative feedback from undergraduate nursing students regarding their experiences with the gamified teaching approach implemented in the Critical Care Nursing course. The questionnaire focused on students’ perceptions, emotional responses, suggestions for improvement, and views on the applicability of this teaching method to other subjects.

**Instructions to Participants**

Please answer the following questions based on your learning experience in this course. There are no right or wrong answers. Your responses will be kept confidential and used only for research purposes.

**Open-Ended Questions**

1. **What are your thoughts on the gamified teaching approach in this course?**
   Please describe your overall learning experience, including your level of engagement and any feelings you experienced during the activities (such as interest, excitement, nervousness, stress, fatigue, or boredom).
2. **Do you have any suggestions for the gamified teaching activities?**
   You may comment on aspects such as game rules, duration, difficulty level, variety of activities, or classroom organization.
3. **How would you apply this gamified teaching method to other subjects?**
   Please explain whether you would like to see this method used in other nursing or medical courses, or why you would choose not to apply it.

**Descriptive Analysis Note**

The open-ended responses were used as descriptive feedback rather than as a formal qualitative research strand. The research team reviewed responses and grouped recurring content into descriptive, non-mutually-exclusive categories. Counts were summarized using the intervention-class denominator (n=65). A participant response could contribute to more than one category, so category counts may overlap and are not expected to sum to 65. No claim of thematic saturation or formal thematic analysis was made.
