# Supplementary material for: Educational outcomes of CDIO-guided progressive case-based learning and gamification in undergraduate critical care nursing: an exploratory two-class quasi-experimental study
Source: Front Public Health. 2026 Oct 9;14:1958746. doi: 10.3389/fpubh.2026.1958746 (PMC13690949; doi:10.3389/fpubh.2026.1958746)
Supplement: Supplementary file 2 [file Table_1.docx]

| **Supplementary Table S1.** Progressive Case-Based and Gamified Learning Program Organized by the CDIO Framework | | | | | |
| --- | --- | --- | --- | --- | --- |
| **CDIO Phase** | **Learning Objective** | **Content and Learning Activities** | **Teaching Strategies** | **Assessment** | **Course Stage** |
| Conceive | Master basic first aid knowledge | Develop micro-lecture videos covering critical care nursing, prehospital emergency care, emergency department resuscitation, and intensive care protocols. Topics include cardiac arrest, acute poisoning, disaster response, severe trauma, and environmental or chemical injuries. Skills include CPR, bag-valve-mask ventilation, automated external defibrillator use, defibrillator operation, endotracheal intubation, ECG monitoring, and the Heimlich maneuver. | Video lessons | Theoretical examination | Before class |
| Conceive | Familiarize students with common emergency procedures for critical and severe conditions | Develop progressive case-based learning cases based on the 'scenario-question-knowledge + skills' framework and design gamified teaching modules. | Case-based teaching; measurement of self-directed learning ability |  | Before class |
| Conceive | Develop independent learning ability | Distribute micro-lecture videos and progressive case-based learning cases, requiring students to analyze scenarios and develop initial problem-solving approaches. | Case-based teaching |  | Before class |
| Conceive | Clarify learning process and requirements | Publish course instructions and game reward rules. | Gamified learning preparation |  | Before class |
| Design | Check the effect of pre-class preparation and improve learning input | Students freely form five-member small groups based on the structure of first aid teams and complete teaching games according to established rules. | Gamification in teaching | Measurement of learning engagement | Theoretical class |
| Design | Consolidate theoretical knowledge of first aid | Teachers reinforce weak knowledge points according to game results. | Multimedia teaching; theoretical assessment |  | Theoretical class |
| Design | Cultivate clinical thinking ability | Teachers guide students to design emergency rescue plans for critically ill patients according to first aid procedures and clarify key operations. | Case-based teaching; measurement of critical thinking ability |  | Theoretical class |
| Design | Develop teamwork and communication skills | Students discuss and improve rescue plans in groups. | Inquiry-based teaching |  | Theoretical class |
| Implement | Practice basic first aid skills | Teachers establish game levels based on progressive scenario cases. Students divide roles according to rescue plans, identify task clues, solve level challenges, perform key operations, and complete scenario drills. | Gamification in teaching; scenario-based simulation teaching | Evaluation of teaching satisfaction; scoring for scenario-based exercises | Laboratory session |
| Implement | Enhance teamwork and adaptability | Students cooperate to solve emergency situations presented in progressive cases. | Scenario-based simulation teaching |  | Laboratory session |
| Implement | Cultivate humanistic care qualities | Students apply communication and patient-centered approaches during scenario activities. | Role-playing method |  | Laboratory session |
| Operate | Strengthen skills in emergency medical procedures | After completing scenario drills, each student performs an individual first aid operation assessment. | Demonstration method | Operation assessment | Laboratory session |
| Operate | Build professional responsibility | Students conduct group drills, reflections, and presentations to share learning experiences. | Report method; peer evaluation and teacher feedback |  | Laboratory session |
| Operate | Improve communication skills | Certificates and small gifts are awarded to outstanding groups and individuals. | Recognition and feedback |  | Laboratory session |
|  | | | | | |

| **Supplementary Table S2.** Design and Implementation of Gamified Teaching Activities for Acute Organophosphorus Pesticide Poisoning | | | | | | |
| --- | --- | --- | --- | --- | --- | --- |
| **Section** | **Learning objective** | **Teaching content** | **Specific implementation process** | **Scoring rules** | | **Teaching link** |
| Overall instructional objectives | Overall objective | Master fundamental knowledge of acute organophosphorus pesticide poisoning; understand emergency assessment and rescue procedures; develop clinical reasoning, teamwork, and emergency response skills; improve application of theoretical knowledge in clinical practice. |  |  | |  |
| Definitely Win Treasure Island | Master basic knowledge of acute organophosphorus pesticide poisoning | Causes, pathogenesis, clinical manifestations, diagnosis, and emergency management of acute organophosphorus pesticide poisoning. | Students worked in teams and progressed through the treasure island activity by answering clinical knowledge questions. Correct answers allowed advancement, while incorrect answers were discussed and corrected. | Points were awarded according to question completion and correctness. Accumulated scores were used for team ranking. | | Theoretical course |
| Definitely Win Treasure Island | Consolidate understanding of poisoning identification and management | Recognition of toxic exposure, symptoms, severity assessment, and initial emergency response. | Teams analyzed scenario-based questions and applied theoretical knowledge to clinical decision-making during the activity. | Scores were calculated based on completed tasks and correct responses. | | Theoretical course |
| Escape-room simulation | Practice basic first aid skills | Identification and management of acute organophosphorus pesticide poisoning, including emergency assessment and first aid procedures. | Students completed sequential clinical scenarios involving poisoning identification, emergency assessment, treatment selection, and rescue procedures. Teams collaborated to solve clinical challenges and progress through escape-room stages. | Completion of scenario tasks contributed to team progression. Performance was evaluated according to task completion, clinical decision-making, and teamwork. | | Experimental lesson |
| Escape-room simulation | Enhance teamwork and adaptive skills | Emergency rescue procedures, communication, teamwork, and application of nursing knowledge in acute poisoning scenarios. | Students analyzed evolving clinical situations, performed decision-making tasks, and completed scenario-based challenges in emergency response teams. | Team performance was evaluated according to completion of rescue tasks, collaboration, and application of clinical knowledge. | | Experimental lesson |
| Escape-room simulation | Cultivate clinical judgment and comprehensive nursing abilities | Integration of assessment, treatment planning, nursing interventions, patient education, and prevention strategies. | Students reflected on clinical reasoning, discussed solutions, and received instructor feedback after completing escape-room activities. | Final performance incorporated task completion, clinical reasoning, and reflection outcomes. | | Experimental lesson |
| *FootNote: The gamified teaching intervention consisted of two components: Definitely Win Treasure Island (a knowledge-based board-game activity) and escape-room simulation (a scenario-based clinical skills activity). Both activities were integrated into the CDIO-guided progressive case-based learning framework. Scoring rules were used for team progression and performance evaluation during gamified activities.* | | | | | | |
|  | | | | |  |  |

| **Supplementary Table S3.** Progressive Case-Based Escape-Room Simulation Design for Acute Organophosphorus Pesticide Poisoning | | | | | |
| --- | --- | --- | --- | --- | --- |
| **Clinical scenario progression** | **Student challenges and questions** | **Knowledge and skills objectives** | **Game implementation process** | **Instructor role and feedback** |  |
| A 56-year-old patient was admitted with drooling and convulsions after pesticide exposure and was transported for emergency care. | Students identify the poisoning cause and explain symptoms through clinical clues. | Knowledge: causes, exposure routes, pathophysiology. Skills: emergency communication and toxic exposure identification. | Five-member teams analyze clues and complete the first challenge to progress. | Instructor observes teamwork, evaluates reasoning, and provides feedback. |  |
| Progressive information reveals unconsciousness, contaminated clothing, empty pesticide bottle, hypotension, secretions, tremors, and other severity indicators. | Students assess disease severity and emergency priorities. | Knowledge: manifestations and severity assessment. Skills: physical examination and emergency assessment. | Teams interpret progressive clinical information and complete assessment tasks. | Instructor provides clarification and reinforces assessment principles. |  |
| Blood cholinesterase activity is measured and found to be less than 0.200. | Students interpret laboratory results and connect findings with diagnosis. | Knowledge: cholinesterase activity interpretation. Skills: diagnostic reasoning. | Teams solve the diagnostic challenge using clinical and laboratory information. | Instructor evaluates reasoning and corrects misunderstandings. |  |
| Patient receives decontamination, medication treatment, ECG monitoring, and cholinesterase monitoring. | Students explain treatment purposes and nursing considerations. | Knowledge: emergency treatment principles. Skills: gastric lavage, medication administration, venous access, ECG monitoring. | Teams complete treatment-related tasks by matching problems with interventions. | Feedback focuses on treatment rationale and emergency priorities. |  |
| Patient develops respiratory arrest during resuscitation and emergency rescue is initiated. | Students identify deterioration causes and rescue priorities. | Knowledge: complications and rescue management. Skills: respiratory support and emergency response. | Teams complete rescue challenges according to clinical priorities. | Instructor evaluates teamwork and decision-making. |  |
| Patient recovers after treatment and nursing care and is discharged. | Students develop prevention and health education strategies. | Knowledge: prevention and health guidance. Skills: patient education and communication. | Teams complete the final challenge by presenting prevention strategies. | Instructor reviews presentations and guides reflection. |  |
| *Footnote: The escape-room simulation was conducted using a progressive case-based design. Students participated in five-member teams and completed sequential clinical challenges. Before activities, instructors introduced learning objectives, scenario background, activity rules, and scoring criteria. Teams progressed through stages according to task completion. Instructor feedback and group reflection were conducted after completion to consolidate clinical reasoning, teamwork, and emergency nursing skills.* | | | | | |

| **Supplementary Table S4.** Game Elements and Implementation Characteristics of the Gamified Teaching Intervention | |
| --- | --- |
| **Game element** | **Description in the present study** |
| Game format | Board-game activities and escape-room simulation integrated into the CDIO-guided progressive case-based learning framework |
| Learning objectives | To reinforce critical care nursing knowledge, clinical reasoning, emergency response skills, teamwork, and communication abilities |
| Team organization | Students formed small teams of five members based on the structure of an emergency response team |
| Competition mechanism | Teams competed through completion of learning tasks, question answering, operational challenges, and overall performance scoring |
| Game rules | Students progressed through predefined game stages according to established rules for each activity |
| Scoring system | Points were awarded based on correct answers, task completion, operational performance, teamwork, and stage completion |
| Rewards | Certificates and small gifts were provided to outstanding teams and individuals based on final performance ranking |
| Feedback mechanism | Teachers provided feedback based on student performance and reinforced weak knowledge points identified during activities |
| Reflection/debriefing | Students participated in group reflection, presentations, and instructor feedback after scenario-based activities |
|  | |

| **Supplementary Table S5.** Intervention Delivery Checklist for the CDIO-Guided Progressive Case-Based Learning and Gamification Program | | |
| --- | --- | --- |
| **Intervention component** | **Planned implementation** | **Delivery status** |
| CDIO framework | Teaching activities organized according to the four CDIO phases: Conceive, Design, Implement, and Operate | Implemented |
| Progressive case-based learning | Progressive clinical cases were delivered according to predefined scenarios and learning objectives | Implemented |
| Team-based learning | Students participated in five-member emergency response teams | Implemented |
| Gamified learning elements | Board-game activities incorporated competition, scoring, task completion, and rewards | Implemented |
| Escape-room simulation | Students completed sequential clinical challenges based on critical care scenarios | Implemented |
| Clinical skills practice | Students performed scenario-related nursing skills and emergency response tasks | Implemented |
| Instructor guidance and feedback | Teachers monitored activities, reinforced weak knowledge points, and provided feedback | Implemented |
| Reflection activities | Students participated in group reflection and presentations after activities | Implemented |
| Assessment procedures | Learning outcomes were evaluated using the predefined assessment methods | Implemented |
|  | | |
